# The Diagnostic Potential of the L Score for ABO Hemolytic Disease of the Newborn: Insights from a Cross-Sectional Study

**DOI:** 10.1007/s12288-023-01723-5

**Published:** 2024-01-05

**Authors:** Yike Li, Jun Deng

**Affiliations:** 1 Department of Clinical Laboratory, Changsha Hospital for Maternal and Child Health Care Affiliated to Hunan Normal University, Changsha, 410007 Hunan China; 2 Department of Clinical Laboratory, The Affiliated Changsha Central Hospital, Hengyang Medical School, University of South China, Changsha, 410004 Hunan China

**Keywords:** ABO hemolytic disease of the newborn, L score, Differential diagnosis, Blood routine examination, Early diagnosis

## Abstract

**Purpose:**

This study aimed to evaluate the diagnostic efficacy of the L score, a novel scoring system, in distinguishing between ABO hemolytic disease of the newborn (ABO-HDN) and non-hemolytic disease of newborn hyperbilirubinemia (NHDNH).

**Methods:**

A cross-sectional prospective study was conducted to assess the effectiveness of the L score in distinguishing between ABO-HDN (*n* = 118) and NHDNH (*n* = 213). Blood routine examination results were collected, and relevant statistical analyses were performed to identify clinically significant parameters. Binary logistic regression analysis was employed to assess the relationship between the L score and the development of these conditions, considering relevant variables.

**Results:**

Our study identified the red blood cell count, mean corpuscular volume, red blood cell distribution width—coefficient of variation, and red blood cell distribution width—standard deviation as independent risk factors for distinguishing ABO-HDN from other high bilirubinemia conditions (*P* < 0.001). The L score demonstrated superior predictive performance for ABO-HDN, exhibiting an area under the curve (AUC) of 0.746, with an optimal cutoff value of − 3.0816. The RBC-L score exhibited superior predictive performance (z: 5.596, *P* < 0.0001) compared to the single-factor RBC indicator, indicating its efficacy in accurately identifying the desired outcome.

**Conclusion:**

The L score represents a valuable tool for predicting neonatal hyperbilirubinemia and hemolytic disease, facilitating differentiation, and guiding early intervention for improved outcomes. Further research is warranted to validate and expand the applicability of the L score in clinical practice.

**Supplementary Information:**

The online version contains supplementary material available at 10.1007/s12288-023-01723-5.

## Introduction

Hemolytic disease of the newborn (HDN) is a common type of neonatal hyperbilirubinemia (NH) that affects approximately 42.6% of infants [1]. In this disease, ABO blood group incompatibility is the most common [2], and its symptoms are milder than Rh hemolysis [3], but there is still the possibility of nuclear jaundice [4]. Therefore, early intervention treatment has important clinical significance. ABO hemolytic disease of the newborn (ABO-HDN) always occurs in the offspring of blood group O mother and newborns born to mothers with blood type A or B [5, 6]. The pathogenesis of ABO-HDN resulting from ABO incompatibility remains unclear, but it is widely accepted that maternal antibodies directed against fetal erythrocytes due to ABO incompatibility initiate erythrocyte destruction, leading to the release of excessive bilirubin [7–9].

Timely detection and treatment of ABO-HDN are crucial in reducing hyperbilirubinemia and the risk of bilirubin encephalopathy, which can cause severe complications such as hearing impairment, developmental delays, and bilirubin encephalopathy [10, 11]. Currently, the neonatal hemolysis test is the only laboratory diagnostic measure available to diagnose ABO-HDN. However, the accuracy of this test can be affected by human factors, and variations in elution methods can alter the detection rates of positive results [12]. Therefore, a combination of neonatal hemolysis tests and other diagnostic tests is essential for accurate and timely diagnosis of ABO-HDN.

It requires specific parameters to distinguish ABO-HDN from other types of hyperbilirubinemia. Blood routine examination is commonly used to diagnose clinical conditions such as anemia, thrombocytopenia, and neutropenia, often observed in NH, and it has lots of specific indicators. Thus, Our study aims to evaluate the effectiveness of these indicators of blood routine examination in the screening of neonatal hemolytic disease. This cross-sectional prospective study will use binary logistic regression analysis to identify independent risk diagnostic factors in the blood routine of neonatal hemolysis patients, comparing them with the non-hemolytic disease of newborn hyperbilirubinemia (NHDNH). The analysis process involves fitting multiple diagnostic factors and constructing a joint prediction model to explore its clinical value in identifying ABO-HDN patients among many cases of neonatal jaundice. The study introduces the L score to simplify the joint prediction model [13, 14]. The study's findings will help improve clinical diagnosis and treatment outcomes, enabling early and accurate diagnosis and timely intervention to prevent complications.

## Materials and Methods

### Ethical Considerations and Consent to Participate

The retrospective analysis of clinical patient data for this study adhered to rigorous ethical standards. Approval for the study was obtained from the Ethics Committee of the Affiliated Changsha Central Hospital, with the reference number 2022-S0097. Sample collection procedures were carried out by the Clinical Laboratory at the Affiliated Changsha Central Hospital, Hengyang Medical School, University of South China. These procedures strictly followed the guidelines outlined in the laboratory's sample collection manual. Additionally, it's worth noting that the inspection department of the laboratory holds ISO15189 certification, underscoring its commitment to maintaining high-quality and ethical standards in sample collection and analysis. Informed consent was obtained from all participants, and the study was conducted with utmost consideration for patient privacy and ethical principles, by international standards and local regulations.

### Study Design and Subjects

This study adopts a cross-sectional observational design, aimed at retrospectively analyzing data from a well-defined cohort of neonates. The primary objective is to elucidate disparities in blood routine parameters between neonates diagnosed with ABO-HDN and those with NHDNH. No intervention or clinical trials were conducted during this investigation. The study solely relies on the retrospective analysis of existing data to draw comparisons and identify independent diagnostic factors, employing binary logistic regression analysis. The study population consisted of 422 neonates who were tested for neonatal hemolysis in three tests at the Laboratory Department of Changsha Central Hospital, affiliated with Nanhua University in Hunan Province.

All participants exhibited hyperbilirubinemia, as confirmed by medical records and outpatient diagnosis records. From the initial cohort, 414 neonates had blood drawn for a blood routine test on the same day, while 83 cases with suspicious results in the three hemolysis tests were excluded from the analysis. The remaining 331 infants were divided into two groups (ABO-HDN and NHDNH) based on clinical symptoms and the results of the three hemolysis tests. The study then aimed to analyze the differences in blood routine indicators between the two groups and identify diagnostic indicators that can distinguish between ABO-HDN and NHDNH. Additionally, the final judgment indicators were used to assess the accuracy of detecting true positive results among the excluded suspicious groups.

Demographic and clinical data, including gestational age, birth weight, gender, mode of delivery, and presence of neonatal morbidity, were collected and recorded for all neonates. Laboratory test results, such as blood type, Coombs test, and elution test, were also documented for further analysis. The routine blood parameters, including White Blood Cell (WBC) count, Red Blood Cell (RBC) count, Neutrophil (NEUT) count, Lymphocyte (LYMPH) count, Mean Corpuscular Volume (MCV), Mean Corpuscular Hemoglobin (MCH), Red Cell Distribution Width-Coefficient of Variation (RDW-CV), and Red Cell Distribution Width-Standard Deviation (RDW-SD), were investigated as diagnostics potential indicators for predicting the risk of hemolytic disease in this scientific study.

### Inclusion and Exclusion Criteria

We established inclusion and exclusion criteria to identify the neonatal hemolytic disease group and the NHDNH group. Neonates were included in the ABO-HDN group if they exhibited early and worsening jaundice, and tested positive for antibody release with incompatible blood types between the mother and baby, regardless of direct antiglobulin test "Anti-yin" and "Anti-yang” results. In HDN disease, "Anti-yin" and "Anti-yang" antibodies arise due to blood group disparities between the mother and baby. "Anti-yin" specifically targets the baby's red blood cells, while "anti-yang" focuses on the mother's red blood cells. When there are mismatches in blood types, these antibodies can trigger the breakdown of the baby's red blood cells, a condition known as hemolysis. The Coombs test is employed to identify and understand the role of these antibodies in neonatal hemolytic disease [15]. We included neonates in the NHDNH group if they had any one of the following criteria: jaundice appearing within 24 h after birth, total serum bilirubin level reaching the phototherapy intervention standard for the corresponding day of life and the corresponding risk factors, or exceeding the 95th percentile of the hour-specific bilirubin risk curve, or bilirubin daily increase exceeding 85 μmol/L (5 mg/dL) or hourly increase exceeding 0.5 mg/dL, long duration of jaundice, with full-term infants over 2 weeks and premature infants over 4 weeks, recurrence of jaundice after subsiding, serum conjugated bilirubin level > 34 μmol/L (2 mg/dL) [16], and obtaining negative results for all three tests assessing neonatal hemolysis. Importantly, any of the aforementioned indicators, in addition to negative results for all three tests assessing neonatal hemolysis, were considered for inclusion in the NHDNH group. We excluded cases with suspected results for the neonatal hemolysis test but with high bilirubinemia.

### Plasma Preparation

To prepare plasma for laboratory testing, 3 mL blood samples were collected from all neonates in both study groups following standard procedures. The blood specimen was centrifuged at 3000 rpm for 3 min, and the resulting plasma was carefully aspirated and transferred to a clean glass test tube for free testing. The remaining red blood cells were washed four times with phosphate-buffered saline, and the eluate was discarded for later use. A 200u1 aliquot of the last eluate was kept in a clean glass tube for reference in case of abnormal diffusion test results. Next, 0.8% suspended red blood cells were prepared by adding 8 u1 of washed red blood cells to 1 mL of normal saline in a separate tube for neonatal blood type and direct antihuman globulin testing. The effluent was also prepared using the heat dissipation method, whereby 400 u1 packed red blood cells were mixed with 400 u1 of normal saline, placed in a 56 °C water bath, and incubated for 10 min with intermittent shaking. After incubation, the mixture was centrifuged at 3000 rpm for 3 min, and the supernatant was collected in a clean test tube for future use.

### Blood Type and Three Tests of Neonatal Hemolysis

In this study, we used the micro-column gel newborn blood typing test card to determine ABO and Rh blood types and confirmed the results of direct antihuman globulin testing (Changchun Boshun Biotechnology, China). Laboratory tests were performed using the Hamilton fully automatic blood type analyzer, TD-A type centrifuge for blood typing serology, and FYQ-type immune microcolumn incubator for incubating micro-column gel cards. Further confirmation of the free antibody test and antibody release test was carried out using one micro-column gel anti-human globulin card. A free antibody test was performed by adding 0.8% A, B, and 0 standard red blood cells to each of 50u1 of the three wells on the left in turn, and then adding 50 u1 of spare neonatal plasma to the three reaction wells respectively. Similarly, the antibody release test was performed by adding 0.8% A, B, and 0 standard red blood cells to each of 50u1 of the three wells on the right in turn, and then adding 50u1 of release liquid to the three reaction wells respectively. After adding the sample, the irregular antibody screening card is placed in the incubator and incubated at 37 °C for 15 min. Following this, perform centrifugation using a special cassette serological centrifuge (900 rpm for 2 min, 1500 rpm for 3 min) to observe the results. The results of different tests for detecting hemolytic disease of the newborn (HDN) are presented in Supplemental Table S1, along with their interpretations and significance. In cases where the direct antiglobulin test, free antibody test, and antibody release test were all positive, the presence of ABO-HDN due to ABO incompatibility was confirmed. Importantly, if only the diffusion test yielded a positive result, the presence of HDN could be determined regardless of the results of the other two tests (positive or negative). It is important to note that negative results in the antibody release test, along with either positive results in the direct antiglobulin test or in the free antibody test, raise suspicion for HDN. Finally, if all three tests were negative, NH to ABO incompatibility was ruled out. All laboratory tests were conducted according to the manufacturer's instructions and carried out by trained personnel who were blinded to the clinical data.

### Statistical Analysis

Statistical analysis was performed using SPSS version 19.0 software (version 19.0, Chicago, Illinois, USA) and GraphPad Prism 5.0 software (GraphPad Software Inc., La Jolla, CA, USA) was used for generating graphs. Continuous variables were expressed as mean ± standard deviation or median (interquartile range), and categorical variables were expressed as frequency (percentage). For the retrospective analysis, the chi-square test was used to compare count data between groups, and the t-test was used to compare continuous data between groups. Binary logistic regression analysis was used to identify clinically meaningful indicators, and multivariate binary logistic regression analysis was used to establish a joint prediction model and L score, by corresponding beta coefficients, standard errors, *p*-values, and odds ratios (OR) with their respective confidence intervals (CI). The receiver operating characteristic curve (ROC) was constructed to determine the optimal critical value, and the area under the ROC curve (AUC), sensitivity, and specificity were calculated using MedCalc software for Windows, version 19.4 (MedCalc Software, Ostend, Belgium) [13, 17, 18]. The significance level was set at 0.05.

## Results

### Participant Characteristics and Hematological Parameters Analysis

Table 1 presents the demographic and serological characteristics of the study participants in both NHDNH and ABO-HDN groups. A total of 213 cases were included in NHDNH 118 cases were included in ABO-HDN, and 83 cases were excluded due to suspicious hemolysis triad test results. The NHDNH group had 130 male and 83 female infants, while the ABO-HDN group had 57 male and 61 female infants (*P* = 0.025). The ABO blood type distribution was significantly different between the two groups, with NHDNH having 89 cases of type A, 91 cases of type B, 31 cases of type O, and 2 cases of type AB, and ABO-HDN having 74 cases of type A, 44 cases of type B, 0 cases of type O, and 0 cases of type AB (P < 0.001). The average age of infants was significantly lower in the ABO-HDN group (2.89 ± 2.23 days) than in the NHDNH group (4.83 ± 3.17 days) (*P* = 0.0281). In terms of hematological parameters, the ABO-HDN group had a significantly higher white blood cell count than the NHDNH group, with a higher neutrophil count and lower lymphocyte count in ABO-HDN. The ABO-HDN group also had a significantly lower RBC count than the NHDNH group, along with higher mean corpuscular volume, mean corpuscular hemoglobin, RBC distribution width-coefficient of variation, and RBC distribution width-standard deviation (*P* < 0.001).

**Table 1:** Demographic and serological characteristics of non-hemolytic disease of the newborn hyperbilirubinemia (NHDNH) and ABO hemolytic disease of the newborn (ABO-HDN) patients

Parameters	NHDNH Group (*n* = 213)	ABO-HDN Group (*n* = 118)	*P*-value
*Demographic variant*			
Gender (m/f)	130/83	57/61	0.025
Age (days)	4.83 ± 3.17	2.89 ± 2.23	0.028
ABO type			
A	89	74	< 0.001
B	91	44	
O	31	0	
AB	2	0	
*Serological variant*			
WBC (*10^9^/L)	11.60 ± 3.98	13.64 ± 5.55	< 0.001
RBC (*10^12^/L)	4.75 ± 0.62	4.55 ± 0.61	0.006
NEUT (*10^9^/L)	5.90 ± 3.74	8.33 ± 5.16	< 0.001
LYMPH (*10^9^/L)	4.06 ± 1.26	3.64 ± 1.11	0.003
MCV (fL)	102.47 ± 5.39	104.57 ± 5.25	< 0.001
MCH (pg)	34.95 ± 1.88	35.71 ± 1.94	< 0.001
RDW-CV (%)	15.54 ± 1.39	16.36 ± 1.70	< 0.001
RDW-SD (fL)	58.35 ± 5.64	61.44 ± 6.25	< 0.001

Data are presented as mean ± standard deviation (SD) for continuous variables and as frequency (percentage) for categorical variables

*NHDNH* Non-hemolytic disease of the newborn hyperbilirubinemia, *ABO-HDN* ABO hemolytic disease of the newborn patients, *WBC* White blood cell, *RBC* Red blood cell, *NEUT* Neutrophil, *LYMPH* Lymphocyte, *MCV* Mean corpuscular volume, *MCH* Mean corpuscular hemoglobin, *RDW* CV Red blood cell distribution width—coefficient of variation, *RDW*-SD Red blood cell distribution width—standard deviation

### Risk Factor Prediction of Hemolytic Disease

A logistic regression model was utilized to predict the risk of hemolytic disease in neonates, specifically comparing patients with NHDNH and ABO-HDN. The model incorporated eight serological difference indicators, including WBC count, RBC count, neutrophil count, lymphocyte count, MCV, MCH, RDW-CV, and RDW-SD. Gender, ABO blood type, and age were also included as covariates in the model. Among them, the WBC count, RBC count, neutrophil count, and lymphocyte count should be assigned and then analyzed. (Supplemental Table S2). A binary logistic regression univariate analysis was performed on each indicator, with a test level of *P* < 0.1, and WBC count and ABO blood type were excluded from the covariates. Then other results were retained for multivariate analysis to continue to adjust and eliminate covariates with *P* > 0.1 in the model. The last result was only RBC count, MCV, RDW-CV, and RDW-SD (Table 2). The logistic regression analysis revealed significant associations between these indicators and the risk of hemolytic disease. The regression coefficients were calculated to quantify the impact of each indicator on the risk. Incorporating these variables into the logistic regression model allowed us to effectively predict the risk of hemolytic disease in neonates. This predictive capability is crucial for early identification and implementation of appropriate intervention strategies for affected individuals. The model's performance in predicting risk factors provides valuable insights for clinical decision-making and the management of the neonatal hemolytic disease.

**Table 2:** Selected risk factors for multivariate binary logistic regression analysis

	Univariate			Multivariate			
Indicator	Beta	*P*-value	OR (min–max)	Beta	SE	*P*-value	OR (min–max)
Gender	0.517	0.026	1.676(1.064–2.640)	0.397	0.257	0.123	1.487 (0.899–2.460)
Age	− 0.215	< 0.001	0.806(0.739–0.881)	− 0.091	0.066	0.165	0.913 (0.803–1.038)
WBC*	0.120	0.274	1.128 (0.909–1.399)	–	–	–	–
*RBC*	0.468	0.009	1.596 (1.126–2.264)	0.402	0.196	*0.040*	1.496 (1.018–2.197)
NEUT	0.540	< 0.001	1.717 (1.359–2.169)	0.073	0.166	0.662	1.075 (0.776–1.490)
LYMPH	− 0.253	0.039	0.776(0.610- 0.978)	- 0.044	0.146	0.761	0.957 (0.719–1273)
*MCV*	0.075	0.001	1.078 (1.031–1.127)	0.140	0.083	*0.093*	1.151(0.977–1.355)
MCH	0.221	0.001	1.247 (1.097–1.418)	0.165	0.116	0.155	1.180 (0.939–1.482)
*RDW-CV*	0.357	< 0.001	1.429(1.213–1.682)	0.934	0.331	*0.005*	2.544(1.329–4.871)
*RDW-SD*	0.089	< 0.001	1.093(1.048–1.139)	− 0.238	0.106	*0.024*	0.788 (0.641–0.969)
ABO blood type*		0.170		–	–	–	–
ABO blood type (1)	21.018	0.999	1.343E9				
ABO blood type (2)	20.476	0.999	7.812E8				
ABO blood type (3)	0.000	1.000	1.000				

Significant results are indicated in bold font and italicized

*WBC* White blood cell, *RBC* Red blood cell, *NEUT* Neutrophil, *LYMPH* Lymphocyte, *MCV* Mean corpuscular volume, *MCH* Mean corpuscular hemoglobin, *RDW*—CV Red blood cell distribution width—coefficient of variation, *RDW*—SD Red blood cell distribution width—standard deviation

^*^The ABO blood type and WBC indictors are not significant for the multivariate binary logistic regression analysis

In our comprehensive analysis investigating the potential risk factors and diagnosis of hemolytic disease, we observed significant associations with various indicators. The results are summarized in Table 3, presenting the beta coefficients, standard errors, *p*-values, and OR with their respective CI. Initially, several factors including gender, age, RBC count, NEUT count, LYMPH count, MCV, MCH, RDW-CV, and RDW-SD were identified as significant predictors of hemolytic disease. However, upon adjusting for confounding factors in the multivariate analysis, only RBC count, MCV, RDW-CV, and RDW-SD remained significant predictors. Importantly, we observed that an increase of one unit in RBC count was associated with a 51.6% higher risk of hemolytic disease (OR 1.516; 95% CI 1.044–2.201). Similarly, each unit increase in MCV corresponded to a 30.6% (OR 1.306; 95% CI 1.130–1.508) higher risk. Contrariwise, each unit increase in RDW-CV exhibited a significant positive association with a substantial 261.8% increase in the risk of hemolytic disease (OR 3.618; 95% CI 1.917–6.828). In contrast, each unit increase in RDW-SD was associated with a noteworthy 28.1% decrease in the risk of hemolytic disease (OR 0.719; 95% CI 0.586–0.884). These findings underscore the importance of considering RBC count, MCV, RDW-CV, and RDW-SD as potential indicators for predicting the risk of hemolytic disease. Higher levels of RBC count and MCV are associated with increased susceptibility to hemolytic disease, while elevated RDW-CV levels and lower RDW-SD levels indicate a higher risk. ABO blood type and WBC count did not emerge as significant predictors of hemolytic disease in the multivariate analyses. In conclusion, our findings emphasize the importance of RBC count, MCV, RDW-CV, and RDW-SD, as significant risk factors for hemolytic disease [19]. These factors should be taken into consideration in the risk stratification and management of patients with this condition.

**Table 3:** Adjusted results of multivariate binary logistic regression analysis

Indicator	Multivariate			
	Beta	SE	*P*-value	OR (min–max)
RBC	*0.416*	*0.190*	*0.029*	*1.516 (1.044–2.201)*
MCV	*0.267*	*.074*	< *0.001*	*1.306 (1.130–1.508)*
RDW-CV	*1.286*	*.324*	< *0.001*	*3.618 (1.917–6.828)*
RDW-SD	− *0.329*	*.105*	< *0.001*	*0.719 (0.586–0.884)*
constant	− *30.069*	*6.470*	*0.000*	*0.000*

RBC Red blood cell; MCV, Mean corpuscular volume; RDW—CV, Red blood cell distribution width—coefficient of variation; RDW—SD, Red blood cell distribution width—standard deviation

### Development of L Score

A logistic regression model was utilized to predict the likelihood (L score) of HDN based on four serological predictive factors, namely RBC count, MCV, RDW-CV, and RDW-SD. The L score can be utilized as a more straightforward and accessible tool for risk assessment and management of the HDN. $P=\frac{1}{1+{e}^{-{\text{variants}}}}$

The logistic regression equation was expressed as logit(P) = – 30.069 + 0.416X_1_ + 0.267X_2_ + 1.286X_3_-0.329X_4_. By transforming the logistic equation, the individual's predicted probability equation was obtained as P = 1/[1 + e^(-(-30.069 + 0.416X_1_ + 0.267X_2_ + 1.286X_3_-0.329X_4_))]. If the predicted probability P was greater than 0.5, the newborn was diagnosed with hemolytic disease. However, since the predicted probability cannot be directly observed, the logistic equation was transformed to simplify its calculation. Each coefficient in the model equation was divided by the smallest coefficient, resulting in the L score equation [13, 17]:

$$ {\text{L}} = - {112}.{618} + {1}.{\text{558X}}_{{1}} + {\text{X}}_{{2}} + {4}.{\text{816X}}_{{3}} - {1}.{\text{232X}}_{{4}} $$

### Evaluation of the L Score and its Constituent Variables

**Fig. 1:**
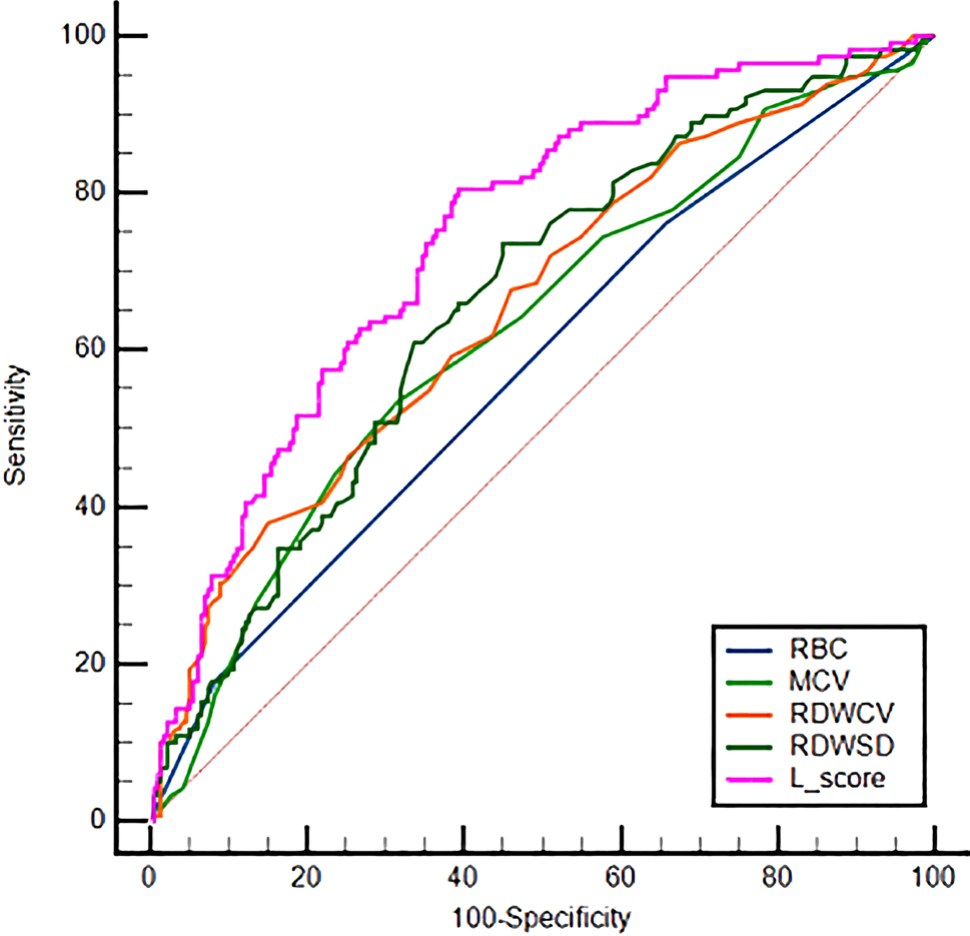
ROC curves predicting neonatal hemolytic disease for L-score and each of the raw covariates. Receiver operating characteristic (ROC) curves were generated to evaluate the predictive performance of the L-score and each raw covariate for neonatal hemolytic disease. The AUC values were calculated to quantify the predictive ability of each predictor. The Figure shows that the L-score had the highest AUC value, indicating that it was the most accurate predictor of neonatal hemolytic disease

Our study aimed to assess the predictive capabilities of the L score and its constituent variables for hemolytic disease in neonates. Table 4 and Fig. 1 present the comprehensive results of our investigation. We utilized ROC curves to evaluate the performance of different indicators. We calculated several performance parameters, including the AUC, optimal cutoff values, sensitivity, specificity, positive likelihood ratio (PLR), negative likelihood ratio (NLR), positive predictive value (PPV), negative predictive value (NPV), and accuracy. Remarkably, the L score demonstrated the highest AUC (0.746) among all tested variables, underscoring its superior predictive performance. The optimal cutoff value for the L score (-3.0816) yielded sensitivity and specificity values of 80.5% and 60.6%, respectively. The calculated PLR (2.041) and NLR (0.322) further support the utility of the L score in identifying high-risk cases. In contrast, individual covariate variables displayed varying AUC values ranging from 0.579 to 0.661, with distinct sensitivity and specificity values. Notably, RDW-SD exhibited the second-highest AUC value (0.661), while RBC had the lowest AUC value (0.579). Our findings emphasize the practicality and accessibility of the L score as a risk assessment tool for managing hemolytic disease. It outperforms individual covariate variables in terms of predictive accuracy. The determined optimal cutoff value of the L score enables accurate identification of neonates at high risk, facilitating timely intervention and treatment.

**Table 4:** Comparison of AUC, optimal cut-off values, and other parameter estimates for each indicator

Variable	AUC	Cut-off value	Sensitivity (%)	Specificity (%)	PLR	NLR	PPV	NPV	Accuracy (%)
RBC	0.579	2	76.3	34.3	1.160	0.692	0.391	0.723	49.2
MCV	0.626	104	53.4	68.5	1.697	0.680	0.485	0.726	63.1
RDW-CV	0.657	16.3	38.1	85.0	2.538	0.728	0.584	0.713	68.3
RDW-SD	0.661	57.8	73.7	54.9	1.636	0.478	0.475	0.791	61.6
L score	0.746	− 3.0816	80.5	60.6	2.041	0.322	0.531	0.849	67.7

*RBC* Red blood cell, *MCV* Mean corpuscular volume, *RDW—CV* Red blood cell distribution width—coefficient of variation, *RDW—SD* Red blood cell distribution width—standard deviation, *AUC* Area under the curve, *PLR* Positive likelihood ratio, *NLR* Negative likelihood ratio, *PPV* Positive predictive value, *NPV* Negative predictive value

### Comparison of ROC Curves

We used Receiver Operating Characteristic (ROC) curves to evaluate and compare the performance of the L score and its constituent variables The results of the ROC curve analysis for different indicators are summarized in Table 5. We determined the statistical significance of each comparison by calculating the z-statistic and corresponding *p*-value. Among the indicators assessed, the RBC-L score demonstrated the highest z-statistic (5.60) and the lowest *p*-value (*P* < 0.0001), indicating its superior predictive capability for the desired outcome. Other indicators, such as the MCV-L score (z-statistics: 3.93; *P* = 0.0001), RDW-CV-L score (z-statistics: 2.91; *P* = 0.0036), and RDW-SD-L score (z-statistics: 3.2; *P* = 0.0014), also exhibited statistically significant differences in their ROC curves. However, comparisons between other scores did not yield statistically significant results. Our analysis revealed that the L score exhibited a statistically significant clinical diagnostic value compared to individual covariate variables [13, 20, 21].

**Table 5:** Comparison of ROC curves for different hemolytic disease predictors

Index comparison	Z-statistic	*P*-value
RBC-MCV	1.279	0.2010
RBC-RDW-CV	1.701	0.0889
RBC-RDW-SD	2.037	0.0416
RBC-L score	**5.596**	** < 0.0001**
MCV-RDW-CV	0.702	0.4829
MCV-RDW-SD	1.365	0.1723
MCV-L score	**3.931**	**0.0001**
RDW-CV-RDW-SD	0.191	0.8486
RDW-CV-L score	**2.913**	**0.0036**
RDW-SD-L score	**3.199**	**0.0014**

*RBC* Red blood cell, *MCV* Mean corpuscular volume, *RDW—CV* Red blood cell distribution width—coefficient of variation, *RDW—SD* Red blood cell distribution width—standard deviation

Significant results are indicated in bold font

### L Score Analyzes 83 Cases Excluded Due to Suspicious Hemolysis Triad Test Results

Additionally, we utilized the collected data to diagnose the suspect groups in three trials using the L score. We assigned and substituted the RBC count, MCV, RDW-CV, and RDW-SD, as significant risk factors for hemolytic disease into the L score formula. Based on the optimal critical value (− 3.0816), a positive result was determined if the value exceeded the critical threshold, indicating the presence of hemolytic disease in the newborn. Conversely, a negative result was assigned if the value fell below the critical threshold. By substituting the results of the original 83 suspicious cases into the L score formula, we diagnosed 47 cases as hemolytic diseases of newborns. According to the positive predictive value of the L score formula, the true positive rate accounted for 53.1%, representing approximately 25 true positive results. The utilization of the L score significantly improves the detection rate of hemolytic disease in the newborn. In conclusion, our findings demonstrate that the L score, along with other relevant indicators, exhibits significant discriminatory power in distinguishing suspected cases. Utilizing the L score formula and determining the optimal critical value allowed us to make accurate diagnoses, leading to improved detection rates for hemolytic disease in newborns.

## Discussion

The main objective of this study is to identify independent diagnostic risk factors in the blood routine of ABO-HDN patients through binary logistic regression analysis. These factors will then be compared with those of NHDNH patients. By incorporating multiple diagnostic factors and creating a unified prediction model, the study aims to assess the clinical significance of these factors in distinguishing ABO-HDN patients from neonates with jaundice. The results of this research could have substantial implications for enhancing clinical diagnosis and treatment outcomes. It's important to clarify that this study does not involve a trial or intervention of participants. Rather, it is an observational cross-sectional study designed to analyze existing data from a specific cohort of neonates. Ethical considerations have been meticulously addressed, including obtaining informed consent from the parents or legal guardians of the neonates involved in the study. The study adheres to strict ethical principles and has received approval from the institutional ethics review board, ensuring the welfare, safety, and privacy of the participants by international standards. The findings of this study have the potential to support early and accurate diagnosis of ABO-HDN, which is essential for preventing complications and improving patient outcomes.

The results showed that RBC, MCV, RDW-CV, and RDW-SD had statistical significance and were independent risk factors for distinguishing ABO neonatal hemolytic disease from other non-neonatal hemolytic disease hyperbilirubinemia. Specifically exhibited a significant positive association with a substantial 261.8% increase in the risk of hemolytic diseases also contributing to the differential diagnosis of another disease, such as Hepatitis B Virus-related chronic liver diseases [22] and α-thalassemia [23]. In contrast, ABO blood type and WBC count were not significant predictors of hemolytic disease in multivariate analyses [24, 25]. These findings are crucial for the proper management of hyperbilirubinemia in newborns, as an accurate and efficient diagnosis of neonatal hemolytic disease is necessary. Overall, the results of this study provide valuable information on the risk factors for neonatal hemolytic disease, which can help improve clinical diagnosis and treatment outcomes and prevent complications.

In addition to the independent risk factors identified in this study, our research represents a significant advancement in the field by introducing the L score as a novel tool for distinguishing ABO-HDN from NHDNH. This study pioneers the utilization of the L score, which combines various indicators including assigned RBC, MCV, RDW-CV, and RDW-SD, in the diagnosis of ABO-HDN. A previous study has highlighted the importance of MCV and RDW as significant factors in diagnosing neonatal hemolytic disease [16], which aligns with our findings. However, our study expands the understanding by demonstrating that the RBC assigned by the reference range, can also serve as independent risk factors for distinguishing neonatal hemolytic disease from other non-neonatal hemolytic disease hyperbilirubinemia. This discovery underscores the potential of incorporating these additional parameters into routine blood screening of neonates with hyperbilirubinemia. Furthermore, our study emphasizes the diagnostic utility of the L score as an independent risk factor for ABO-HDN. By comparing its accuracy to the other factors identified in this study, we provide valuable insights into its effectiveness as a diagnostic index. This groundbreaking approach could have significant implications for clinical practice, empowering physicians with a more intuitive basis to assess the patient's condition and make informed clinical judgments. The results presented in this study not only contribute to the existing body of knowledge but also pave the way for future investigations in the field of neonatal hemolytic disease diagnosis. The introduction of the L score as a comprehensive and reliable tool offers immense potential for enhancing diagnostic accuracy and improving patient outcomes. This research not only provides a rigorous scientific foundation but also holds promise for translating into real-world clinical applications.

In addition to the causes of neonatal jaundice and hyperbilirubinemia mentioned in previous studies, such as physiological jaundice, prematurity, breast milk jaundice, G6PD deficiency, thalassemia, and sepsis [1, 26–28], the definitive indicator for diagnosing the neonatal hemolytic disease is the presence of a positive result in the antibody release test, which involves three tests for neonatal hemolysis. However, it is important to note that these tests are susceptible to significant human interference and may not always yield accurate results [12, 29].

To address this issue, the present study examined potential independent risk factors for the diagnosis of neonatal hemolytic disease. Data were collected from infants with hyperbilirubinemia who underwent both blood routine and neonatal hemolytic disease screening at the hospital over 2 years. The findings of our study indicate significant differences in age, gender, and blood type ratios between the neonatal hemolytic disease group and the non-neonatal hemolytic disease hyperbilirubinemia group. However, it is important to note that these factors alone cannot be considered independent risk factors for diagnosing hemolytic disease in newborns. The complexity of neonatal jaundice and the limitations of current diagnostic tests highlight the need for alternative approaches. Our study introduces the L score, which incorporates multiple indicators (RBC, MCV, RDW-CV, and RDW-SD), providing a more comprehensive and objective assessment. By reducing reliance on subjective interpretations and potential human errors associated with traditional antibody release tests, the L score shows promise in improving the accuracy and reliability of neonatal hemolytic disease diagnosis. Further research and validation studies are necessary to establish the clinical utility and broader applicability of the L score in routine practice. This advancement has the potential to enhance patient care and outcomes in the management of neonatal jaundice. Our findings contribute to the existing knowledge and open avenues for future investigations in neonatal hemolytic disease diagnosis. The introduction of the L score as a comprehensive and reliable tool holds promise for improving diagnostic accuracy and translating it into real-world clinical applications.

However, it is crucial to acknowledge the limitations inherent in this study. Firstly, the relatively small sample size utilized raises concerns regarding the generalizability of the findings. The limited number of participants may constrain the ability to draw broader conclusions and extrapolate the results to larger populations. Secondly, the study's single-hospital setting introduces potential limitations in terms of external validity. The outcomes obtained within this specific hospital may not necessarily reflect the characteristics or circumstances present in other healthcare facilities or geographical regions. Thus, caution should be exercised when applying these findings to a broader context. Thirdly, it is important to note that the gold-standard diagnostic methods for neonatal hemolytic disease were not incorporated into this study. The decision to exclude these tests was primarily based on their utilization of the manual microcolumn gel card technique, which is prone to significant human interference and introduces a potential source of bias. Consequently, the absence of these tests may restrict the comprehensive evaluation of hemolytic disease in this particular study. Furthermore, it should be acknowledged that the limitations of the gold standard operation may result in the omission of some truly positive results. However, the L score can serve as a valuable supplementary tool for identifying suspicious results and potentially increasing the detection rate. Each laboratory can establish an L score with a unique equation based on its data. Nonetheless, the independent risk factors selected by the L score in this study can provide a useful reference for other laboratories. These limitations highlight the necessity for further research incorporating larger sample sizes, multi-center settings, and the inclusion of standard diagnostic tests such as the direct Coombs test and the antibody release test. Conducting studies that address these limitations would contribute to a more robust understanding of the prevalence and factors associated with neonatal hemolytic disease, advancing knowledge in this medical field.

Overall, the findings of this study suggest that differences in RBC, MCV, RDW-CV, and RDW-SD in blood routine had statistical significance and can be used as independent risk factors for distinguishing neonatal hemolytic disease from other non-neonatal hemolytic disease hyperbilirubinemia. These findings can help clinical physicians make more accurate diagnoses and better treat newborns with hyperbilirubinemia. Further studies with larger sample sizes and more diverse populations are needed to confirm these findings and improve the diagnosis and treatment of neonatal hemolytic disease.

### Supplementary Information

Below is the link to the electronic supplementary material.

Supplementary file1 (DOCX 26 KB)

## Data Availability

All relevant data are within the manuscript and its Supporting Information files.
