# Supplementary material for: The Diagnostic Potential of the L Score for ABO Hemolytic Disease of the Newborn: Insights from a Cross-Sectional Study
Source: Indian J Hematol Blood Transfus. 2024 Jan 5;40(3):469–78. doi: 10.1007/s12288-023-01723-5 (PMC11246374; doi:10.1007/s12288-023-01723-5)
Supplement: Supplementary file 1 — Supplementary file1 (DOCX 26 KB) [file 12288_2023_1723_MOESM1_ESM.docx]

**Supplementary Material**

**The Diagnostic Potential of the L Score for ABO Hemolytic Disease of the Newborn: Insights From a Cross-Sectional Study**

**Yike Li ^1^, Jun Deng ^2,*^**

1. Department of Clinical Laboratory, Changsha Hospital for Maternal & Child Health Care Affiliated to Hunan Normal University, Changsha, Hunan 410007, China.

2. Department of Clinical Laboratory, The Affiliated Changsha Central Hospital, Hengyang Medical School, University of South China, Changsha, Hunan 410004, China.

*** Correspondence**

Jun Deng, Department of Clinical Laboratory, The Affiliated Changsha Central Hospital, Hengyang Medical School, University of South China, Changsha, Hunan 410004, China.

Tel: +86-13637475140.

E-mail: 2018050749@usc.edu.cn

***Journal of the Indian Journal of Hematology and Blood Transfusion***

**Inventory of Supplementary Information**

- **Supplementary Table S1‎ (Page 1)**
- **Supplementary Table S2‎ (Page 2)**

**Supplementary Table S1.** Interpretation of the results of ABO blood group hemolytic disease of the newborn (HDN) serological testing

| **Direct Antiglobulin Test** | **Free Antibody Test** | **Antibody Release Test** | **Result Interpretation** |
| --- | --- | --- | --- |
| + | + | + | HDN confirmed |
| + | - | + | HDN confirmed |
| - | + | + | HDN confirmed |
| - | - | + | HDN confirmed |
| + | + | - | Suspicious HDN |
| + | - | - | Suspicious HDN |
| - | + | - | Suspicious HDN |
| - | - | - | HDN denied* |
| *Serological testing failed to confirm HDN.  The tests include a direct antiglobulin test, free antibody test, and antibody release test. The significance of each result is determined by whether HDN is confirmed or denied based on the tests, and whether the type of HDN is ABO HDN. | | | |

**Supplementary Table S2.** Assignment of covariate values in the logistic regression model.

| **Covariate** | **Covariate Assignment** |
| --- | --- |
| WBC (*10^9^/L) | 15.00-20.00=0，<5.00=1,5.00-10.00=2，10.00-15.00=3，>20.00=4 |
| RBC (*10^12^/L) | 6.00-7.00=0，>7.00=1，5.00-6.00=2，4.00-5.00=3，<4.00=4 |
| NEUT (*10^9^/L) | 1.80-6.30=0，<1.80=1，>6.30=2 |
| LYMPH (*10^9^/L) | 1.10-3.20=0，<1.10=1，>3.20=2 |
| Abbreviations: WBC, White Blood Cell count; RBC, Red Blood Cell count; NEUT, Neutrophil count; LYMPH, Lymphocyte count.  Note: The covariate assignments presented in the table are the results obtained after assigning values to each covariate in the logistic regression model. The values represent different ranges or thresholds for each covariate. These assignments enable the incorporation of these covariates into the logistic regression analysis, allowing for the assessment of their impact on the risk of hemolytic disease. The assigned values aid in the interpretation and evaluation of the logistic regression model's predictive performance | |
